# Limitations and Extensions of the Lock-and-Key Principle: Differences between Gas State, Solution and Solid State Structures

**DOI:** 10.3390/ijms16046694

**Published:** 2015-03-25

**Authors:** Hans-Jörg Schneider

**Affiliations:** Universität des Saarlandes, FR Organische Chemie, D 66041 Saarbrücken, Germany; E-Mail: h-j.schneider@mx.uni-saarland.de or ch12hs@rz.uni-sb.de; Tel.: +49-681-811183; Fax: +49-681-6852615

**Keywords:** host and guest complexes, supramolecular chemistry, lock-and-key principle, solvent effects, stereoelectronic effects, binding mechanisms, non-covalent interactions, hydrophobic effects, high energy water, crystal structures, crown ethers, cyclophanes, calixarenes, cyclodextrins, cucurbiturils

## Abstract

The lock-and-key concept is discussed with respect to necessary extensions. Formation of supramolecular complexes depends not only, and often not even primarily on an optimal geometric fit between host and guest. Induced fit and allosteric interactions have long been known as important modifications. Different binding mechanisms, the medium used and pH effects can exert a major influence on the affinity. Stereoelectronic effects due to lone pair orientation can lead to variation of binding constants by orders of magnitude. Hydrophobic interactions due to high-energy water inside cavities modify the mechanical lock-and-key picture. That optimal affinities are observed if the cavity is only partially filled by the ligand can be in conflict with the lock-and-key principle. In crystals other forces than those between host and guest often dominate, leading to differences between solid state and solution structures. This is exemplified in particular with calixarene complexes, which by X-ray analysis more often than other hosts show guest molecules outside their cavity. In view of this the particular problems with the identification of weak interactions in crystals is discussed.

## 1. Introduction

After Emil Fischer coined the lock-and-key picture for the reaction between enzymes and substrates [1], it became a leading concept for the understanding of intermolecular interactions with proteins, and later for the rational design of drugs. With the advent of supramolecular chemistry the idea gained an enormous momentum, as chemists began to synthetize a large variety of host compounds for practically all possible target guest molecules occurring in nature or in the environment. Although few concepts have played a comparatively important role in chemistry, the lock-and-key principle has limitations and extensions, which often are overlooked.

## 2. Dependence on the Binding Mechanism/Medium, pH and Stereoelectronic Effects

First of all, there are fundamental differences in the function of the lock-and-key principle in the gas state and in solution; the situation in crystals is again quite different and will be discussed in Section 6 and Section 7. In solution the presence of a geometrically well-fitting cavity in a receptor is not enough for the binding of a substrate: the price for desolvation of the host and guest prior to complex formation must be paid by compensating non-covalent forces between host and guest, although complete desolvation might not be necessary, and desolvation alone can also contribute to a gain in free energy (see Section 5 on hydrophobic effects). Only in fairly rigid molecular containers [2], the inside binding of substrates may be controlled solely by the size of the portals. Obviously, the penalty for desolvation can be so large that one must change the reaction medium in order to achieve efficient complexation; a well-known example is the design of receptors for recognition of carbohydrates in water [3,4]. Furthermore, the geometric requirements for an optimal binding between host and guest differ enormously with the different non-covalent interactions [5]. Coulombic forces, with an r^−1^ dependence of the binding enthalpy on the distance r between interaction atoms or groups, tolerate much more deviation from a perfect geometric fit than for example dispersive interactions, which fall off with r^−6^, and hydrogen bond strength depends significantly on orientation of donor and acceptor.

**Figure 1:**
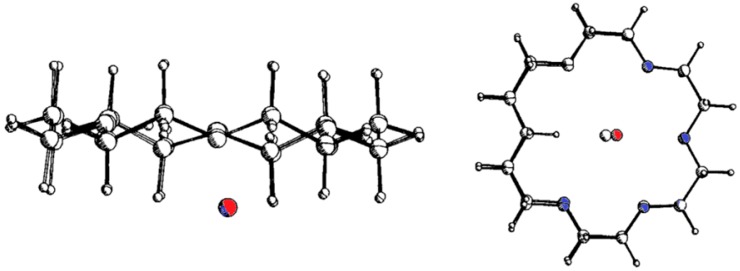
Complexation of potassium ions with crown ethers 18C6 and 18C5; superimposed structures of the K^+^-complexes (the K^+^-ion in the 18C5 complex in red); with binding free energies ΔG in kJ/mol, and differences ΔΔG between them [7]. Adapted with permission from Raevsky, O.A.; Solovev, V.P.; Solotnov, A.F.; Schneider, H.-J.; Rüdiger, V. Conformation of 18-crown-5 and its influence on complexation with alkali and ammonium cations: Why 18-crown-5 binds more than 1000 times weaker than 18C6. *J. Org. Chem.* **1996**, *61*, 8113–8116. Copyright 1996 American Chemical Society.

Solvent effects can be more decisive for complexation strength than size matching. Complexation with crown ethers 18C6 and 18C5 shows that not only the absolute binding energies depend on the medium, essentially as linear function of the cation desolvation free energies of the guest metal ions as shown with a variety of solvents [6]. Also, the differences between 18C6 and 18C5, which binds weaker due to one hydrogen atom protruding into the cavity, are much smaller in water than in other solvents (Figure 1 and Table 1) [7].

**Figure 2:**
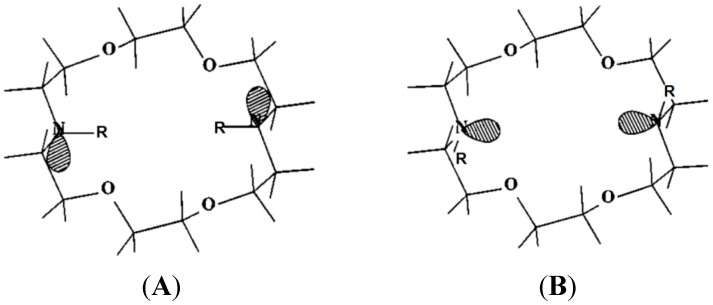
Stereoelectronics: the 1.10-diaza-crown with R = H (diaxial lone pair (lp) orientation, (**A**) binds K^+^ ions with only ΔG = 10 kJ/mol, with R = Me (diequatorial lp orientation; (**B**) ΔG increases to 26 kJ/mol (in methanol) [8].

**Figure 3:**
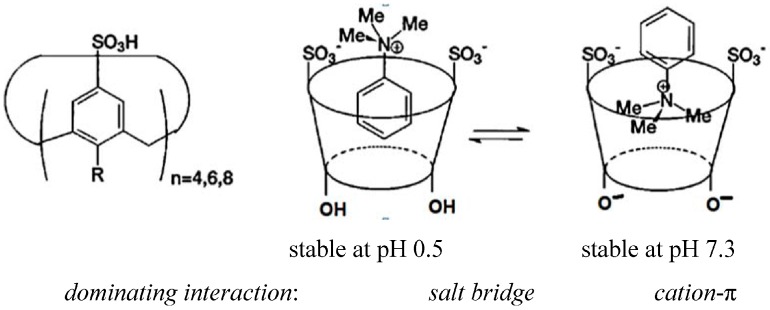
Change of inclusion mode with a calix[4]arene host (*n* = 4) as function of the pH [10].

Stereoelectronics can play a dominating role in complexation strength. A 1.10-diaza-crown ether (Figure 2) binds metal ions much weaker than expected, due to the unfavourable diaxial orientation of the lone pairs (lp) at nitrogen [8]. Introduction of a methyl groups at the nitrogen atoms enforces a diequatorial lp orientation, and the binding energy increases to ΔG values expected for such ionophores [9]. The consequences of a different binding mechanism are illustrated in Figure 3. Here a change in pH alters the inclusion mode of a ligand in the calix[4]arene host, due to a alternatively dominating ion pair or cation-π interaction [10].

**Table 1:** Complexation free energies (in kJ/mol) of crown ethers in different solvents, with differences between 18C6 and 18C5.

	KCl in H_2_O	KCl in MeOH	NaCl in H_2_O	NaCl in MeOH
18C6 ΔG	11.6	34.5	4.6	25.0
18C5 ΔG	7.5	15.9	4.5	14.0
ΔΔG	4.1	18.6	0.1	11.0

**Figure 4:**
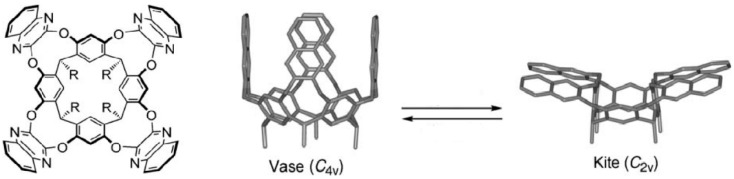
Cavitands which switch between close “vase” and open “kite” conformations [13]. Reprinted from [13] with permission from VCH/Wiley.

Electron densities can play a larger role than geometric fitting. Molecular clips and tweezers bear a highly negative surface potential inside; the binding of the preferred guest molecules such as, e.g., NAD^+^ is therefore dictated more by Coulombic forces than by exact fitting [11]. Ancillary ligands such as tetraaza-cyclododecanes can increase the positive charge at bound highly polarizable lanthanide ions, thereby leading to enhanced sensing affinities towards anions [12]. Cavitands as those shown in Figure 4 exhibit switching between close “vase” and open “kite” conformations as a function of pH, temperature, and of solvent, with the kite preferred in nonpolar solvents [13].

## 3. Induced Fit

**Figure 5:**
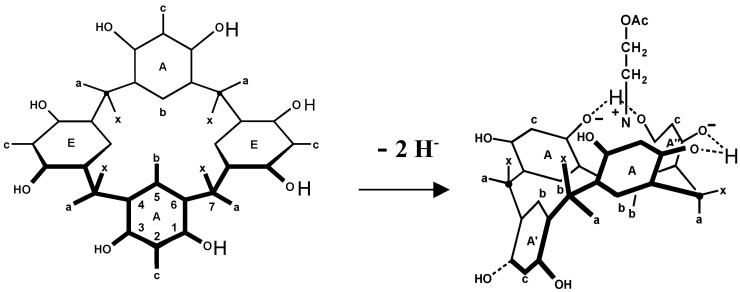
Binding of cholinacetate (Me_3_^+^N(CH_2_)_2_OAc) in a resorcarene macrocycle by induced fit (Me groups at **^+^**N omitted).

An important extension of the lock-and-key principle was introduced early by Koshland, who proposed that conformational changes in an enzyme, induced by the substrate, can strengthen the binding [14]. With synthetic hosts binding is often only possible by severe conformational distortions of the host, as demonstrated e.g., with metalloporphyrin cages [15]. In artificial receptors such an induced fit is particularly obvious if the host is flexible and/or too wide for tight fitting. The resorcarene macrocycle in Figure 5 can bind acetylcholine only in a closed conformation; simultaneously two protons are liberated, thus enabling hydrogen bonds between three phenolic units [16].

**Figure 6:**
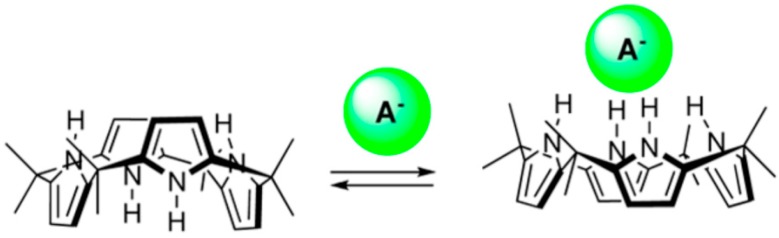
Calix[4]pyrrole in the 1,3-alternate conformation (left side) converts to the cone form by anion binding.

With a calix[6]arene derivative, encapsulation of different charged or neutral species in the hydrophobic cavity is also accompanied by conversion from the 1,3-alternate to the 1,3,5-alternate conformation [17]. Calix[6]arenes possess a particularly high flexibility; their cavity can by induced fit expand for large ligands or shrink for smaller guest molecules [18]. Other examples are calix[4]pyrroles which in solution occur in several conformations, but in presence of anions only in the cone conformation (Figure 6); remarkably one finds in crystals mostly the 1,3-alternate form [19,20].

**Figure 7:**
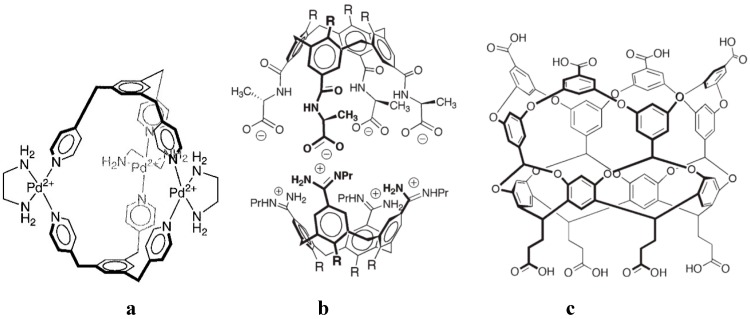
Self-assembly of predesigned host parts to form capsules, (**a**) with adamantanecarboxylic acid as guest [24]; (**b**) by ion pairing, with e.g., *N*-methylquinuclidinium cation as guest [22]; (**c**) a lipophilic host which self-assembles in presence of a long steroid by hydrophobic interactions [25].

Sometimes a host cavity is only formed by inducing with an added guest the self-assembly of predesigned host parts, leading to so-called capsules [21,22,23]. Thus, an assembly of three palladium atoms and two *tris*-pyridyl ligands is induced by adamantanecarboxylic acid (Figure 7a) [24]; a capsule stabilized by ion pairing forms in presence of e.g., *N*-methylquinuclidinium cation as guest [22] (Figure 7b); or a steroid as guest induces a host assembly by hydrophobic interactions [25] (Figure 7c).

## 4. Allosteric Effects

**Figure 8:**
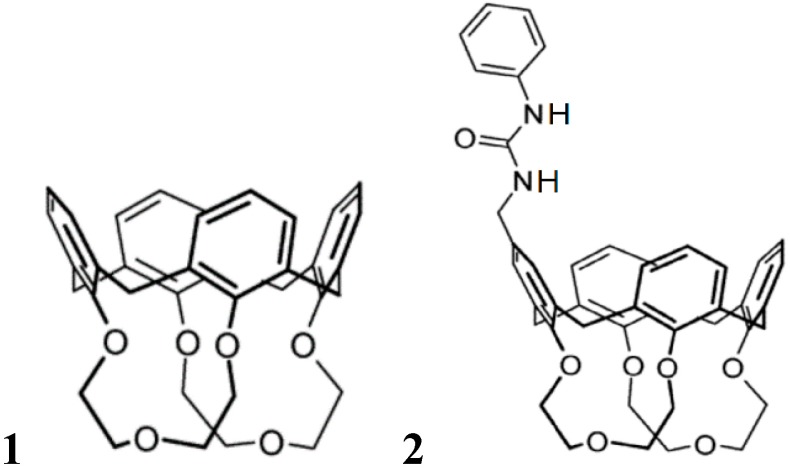
Association constants *K*_as_ (M^−1^) of 1:1 complexes of tetramethylammonium salts Me_4_N^+^·X^−^ with hosts **1** and **2** in CDCl_3_, in presence of tosylate, chloride, acetate or trifluoroacetate anions [30].

An important extension of the simple lock and key concept is due to allosteric interaction of a second guest component which is not directly acting at the first binding site. A large number of synthetic host guest complexes have been designed which show the typical binding modulation by the presence of a second effector [26,27,28,29]. This occurs most often, but not necessarily by conformational changes. Figure 8 and Table 2 illustrates the strong influence of an anion as second effector on the binding strength of tetramethylammonium salts in selected calixarenes. NMR analyses verified that the ammonium group is filling the cavity, so that the anion, which forms a strong ion pair with the cation in the apolar solvent chloroform used here, can only bind outside the calix, particularly efficiently with the urea group in the then heterotopic receptor **2** [30].

**Table 2:** Association constants *K*_as_ (M^−1^) of 1:1 complexes of tetramethylammonium salts Me_4_N^+^·X^−^ with hosts **1** and **2** (Figure 8) in CDCl_3_, in presence of tosylate, chloride, acetate or trifluoroacetate anions.

X	TsO	Cl	OAc	TFA
Host **1**	33	80	250	360
Host **2**	700	8800	5000	13,000

**Figure 9:**
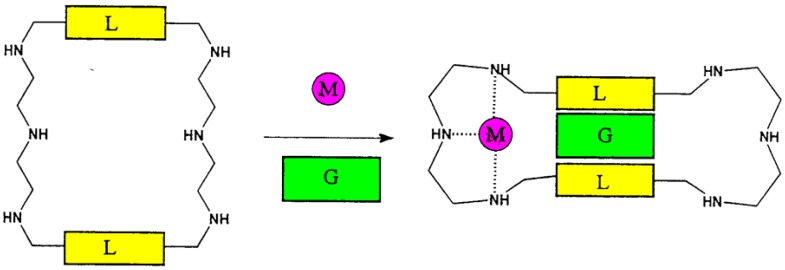
An example of an allosteric system (L = *p*-phenyl, M = Zn^2+^, G = dansylamide) in which introduction of metal ions lead to a ratio of binding constants of K_M_/K_0_ >> 100; fluorescence emission occurs only in presence of metal ion [31,32].

Artificial host compounds can show much stronger allosteric effects than proteins, in which conformational coupling between interacting binding sites is usually much weaker. The example in Figure 9 shows a particularly large ratio K_M_/K_0_ of binding constants with and without second effector; only in the presence of metal ions such as Zn^2+^, a cavity is formed by contraction which binds lipophilic substrates such as dansylamide [31,32].

## 5. Hydrophobic Interactions beyond the Lock-and-Key Picture

**Figure 10:**
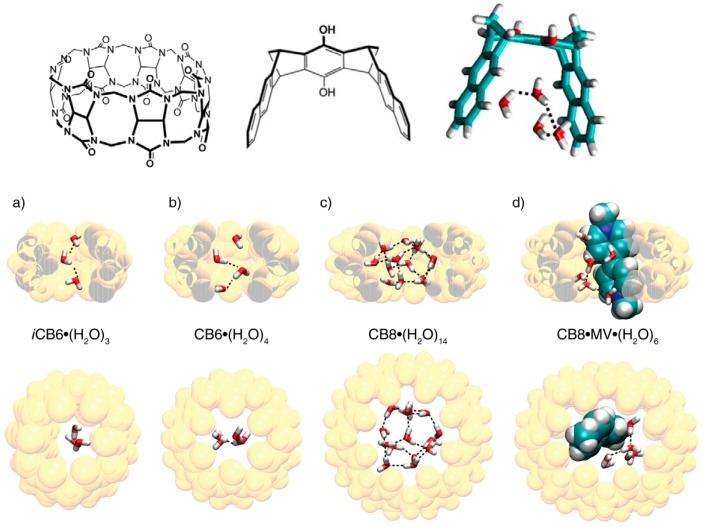
Host compounds for large hydrophobic binding contributions: cucurbiturils and a molecular clip with four water molecules. Cucur[n]biturils with increasing size: (**a**) Crystal structure of inverted-CB6 with three intracavity water molecules; (**b**–**d**) Snapshot from molecular dynamics (MD) simulations for (**b**) CB6, (**c**) CB8 and (**d**) CB8·viologen complexes with 4, 14 and six cavity water molecules, respectively. **Top**: Complexes viewed from the side (CB*n* atoms in the front removed for clarity); **Bottom**: Complexes viewed from the top. Reprinted from [33] with permission from VCH/Wiley.

At first sight it seems that hydrophobic forces, which were traditionally ascribed to an entropy advantage gained by association between lipophilic molecules and subsequent liberation of water molecules, should not lead to particular deviations from the lock-and-key principle: the larger and closer the contact between a host cavity and a guest, the larger will be the number of liberated water molecules. In line with this idea hydrophobic contributions are traditionally evaluated by determination of solvent excluded surfaces. However, there is increasing and recently quantified evidence, that in host guest complexes significant contributions stem from the liberation of high energy water molecules [33,34,35,36] which in cavities can materialize less than the four hydrogen bonds which exist in bulk water [37]. Without complexation in a cavity there is only a very small hydrophobic effect, even for saturated compounds [38]. It has been shown that for essentially closed cavities such as in cucurbiturils the binding free enthalpies with some guest compounds can be completely explained by this non-classical high-energy water effect [33]. This is particularly so if the host interior offers few non-covalent interactions, as is the case for cucurbiturils, but also for some molecular clips (Figure 10). The higher the number of high-energy water molecules is in a cavity, and the smaller the number of hydrogen bonds of each of these water molecules is, the larger is the energy gain; in accordance to the lock-and-key principle this would be achieved if the fit between host and guest is so perfect that all water molecules are displaced by the guest. However, if the host is large enough to accommodate more water molecules which can develop a satisfactory number of hydrogen bonds the hydrophobic driving force will play a minor role even if there is a perfect fit with a large enough guest which displaces all water molecules. Large hosts such as some cucurbiturils can accommodate a guest molecule and water, which again can exert more or less hydrogen bonds, or even two guest molecules. These possibilities are illustrated in Figure 10; complexes with cucurbiturils but also with cyclodextrins or molecular clips exhibit sizeable high-energy water effects [33]. It has been stressed that also the binding affinity in protein pockets is often not dominated by the lock-and-key principle but by the displacement of free-energetically unfavourable water [39,40].

## 6. Host and Guest Complexes in the Solid State

**Figure 11:**
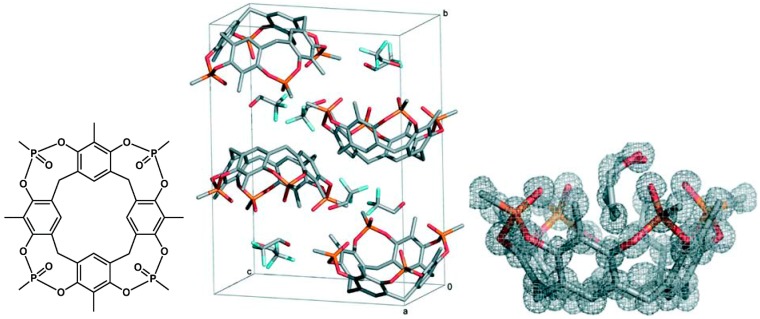
Example of a crystal of a resorcarene cavitand, containing co-crystalizing trifluorethanol, which forms isomorphous structures with a series of linear alcohols; the refined structure with e.g., *n-*propanol as ligand shows the relevant electron densities. Reprinted from [44] with permission of the Royal Society of Chemistry.

In crystals the lattice is stabilized by a multitude of interactions in addition to those between host and guest; the uptake of a guest molecule can lead to a significant change of the solid state structure of the host alone. Metastable different crystalline modifications of the same compound, or polymorphs, are possible in particular if energy differences between molecular conformers and crystal lattice energies are of the same magnitude [41,42]; they are also quite frequent in cocrystals [43]. Occurrence of polymorphs make the assignment of an optimal host-guest geometry more difficult, but can shed light on the different interaction mechanisms. Isomorphic crystals can show a more unified picture of host and guest complexes, if they offer enough room for ligands, particularly if these are relatively small and if the chemical properties as well as binding mechanisms of different ligands are similar. Such conditions are also typical for complexes with large biomolecules such as proteins, in which the receptor conformation is in addition stabilized by a multitude of interactions. Figure 11 presents an example of a crystal which forms isomorphous structures with a series of linear alcohols [44]. Interestingly, crystals of inclusion compounds with the guest inside the cavity can often be obtained simply by slow diffusion of guests into the solvent-filled voids of the crystalline sponges [45], or by exchange of one guest with another one with the complex crystals in the vapour phase [46].

The abovementioned similarity between crystals of one receptor with small guest molecules is also the basis of an interesting new method to test selectivities from occupancy factors in a crystal with competing guest molecules [44]. Thus, isomorphous monoclinic crystals with a resorcarene cavitand and six alcohols were X-rayed without the unnecessary structural refinement; the observed occupancy factors were in close agreement with the relative binding constant ratios of the alcohols. The fully refined structure of the crystal with e.g., *n-*propanol (Figure 11) shows that the small ligand finds its place without significant distortion of the lattice; comparison with the different alcohols shows an affinity decrease with the increase in the host-guest hydrogen bond distance, which is a function of the alcohol chain length.

## 7. Intra- and Extra Cavity Complexation in Macrocyclic Receptors/Differences between Solid State, Gas State and Solution Structure

**Figure 12:**
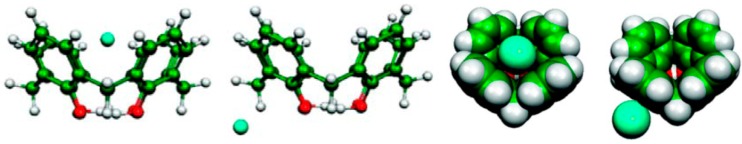
Calix[4]arene complexes with argon; optimized structures of endo-complex and exo-complex. Reprinted from ref. [47,48] with permission of the Royal Society of Chemistry.

The rather shallow cavity of small calixarenes lead particularly often to extra- (or exo-) cavity complexation, although the simple lock-and-key principle would predict an intra- (or endo-) complex. For complexes between argon and calix[4]arene in the gas state, spectroscopic and quantum-chemical calculations show both orientations, as expected with a preference for the endo-complex (Figure 12) [47,48]. Laser spectroscopic molecular beam experiments and computations of calix[4]arene complexes with a variety of small ligands such as NH_3_, N_2_, CH_4_, and C_2_H_2_ indicate also preferred endo complexes, for H_2_O and NH_3_ as guest mainly by dipole–dipole interactions, for Ar, N_2_, CH_4_ and C_2_H_2_ mostly by dispersion forces [49].

**Figure 13:**
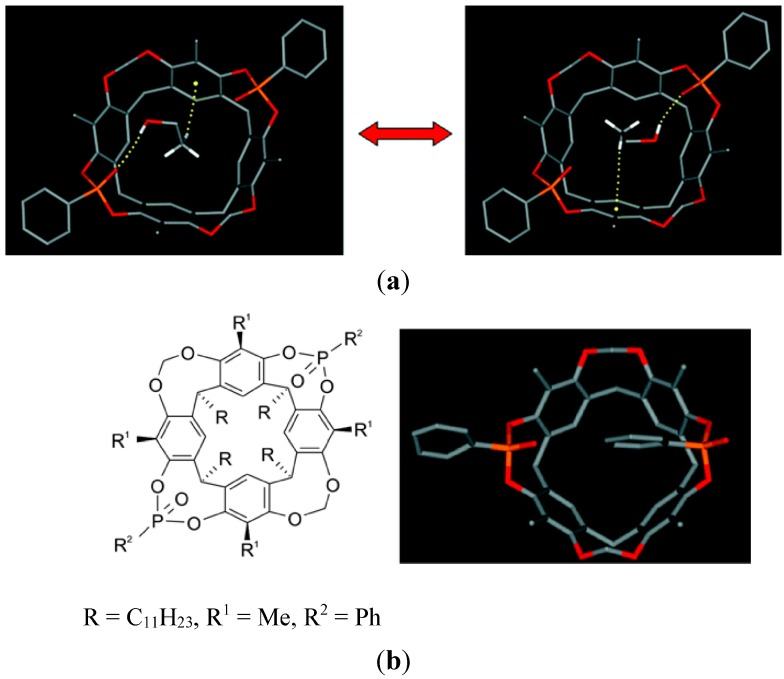
(**a**) Resorcarene complexes with ethanol exhibiting two different structures within one crystal (hydrogen bonds of EtOH with the two distal P=O groups with a 50% statistical probability); (**b**) isomeric structure with a phenyl group filling the cavity; ethanol can only bind outside the cavity [50]. Reprinted from ref. [50] with permission of the Royal Society of Chemistry.

That interactions in the solid state are effective also in the gas phase complexes has been aptly discussed by Dalcanale *et al.* with complexes based on calixarenes or resorcarenes with P=O groups as hydrogen bond acceptors [50]. Electrospray ionization mass spectrometry (ESI-MS) is a suitable technique to elucidate what happens in the gas state. A major difference is that in the gas phase the outward facing P=O groups are not shielded by neighbouring molecules as in the solid layer, and are therefore amenable to H-bonding with the guest. The complex between the resorcarene cavitand and ethanol (Figure 13) is also a nice example of several supramolecular structures within a crystal, exhibiting hydrogen bonds of EtOH with the two distal P=O groups with a statistical 50% probability; one also observes the synergy of P=O···H–O bonding and CH–π interactions in the cavitand (Figure 13a). If as in an isomeric structure (Figure 13b) a phenyl group fills the cavity, no C–H···π interaction is possible and also no H-bond to the then outward P=O group; then ethanol is found outside in the crystal lattice. For solid receptor layers, used often for gas detection, the distinction between intracavity *vs.* extracavity complexation is a particular problem. Location of analytes in the receptor layers can be identified by FT-IR spectroscopy if host and guest diagnostic bands do not overlap due to unspecific adsorption. Unspecific adsorption is characterized by linear adsorption isotherms, in contrast to Langmuir-type isotherms, which deviate significantly from linearity, indicating a specific analyte-layer interaction.

Complexes with smaller calixarenes show relatively often guest binding outside the cavity, as found e.g., in crystals of the calix[4]arene with toluene; here the guest molecule occupies intermolecular cavities of host channels [51]. In solution amines in the form of ammonium ions bind to calixarenes or resorcarenes usually as intracavity complexes [52,53], essentially due the cation-π interaction. In the solid state, however, amines bind often to the exo side, or to both sides. Thus, *p*-*tert*-butylcalix[4]arene forms with 1,4-butanediamine an inclusion compound with amine side both exo and endo of the cavity [54]. Both orientations were also found for complexes of amines and calix[6]arene [55]. In a *p-tert*-butylcalix[7]arene 1:3 pyridine crystal two pyridines have been found outside the cavity in the crystal lattice, forming a complex/clathrate hybrid [56]. Crystals of *p-tert*-butylcalix[8]arene with 8 pyridine molecules in the unit cell show the host macrocycle in an open chairlike conformation, so there is no cavity for the guest molecule [57].

**Figure 14:**
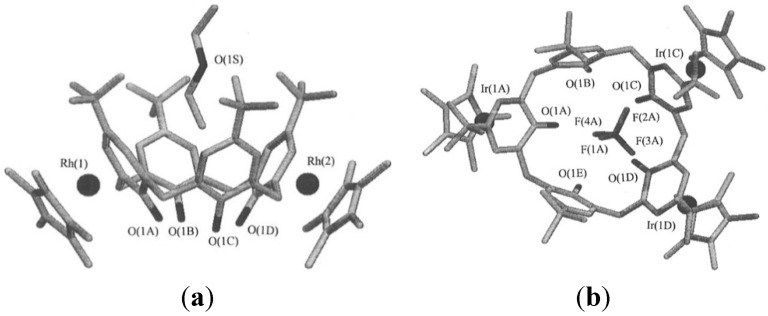
(**a**) Crystal structure of a dirhodium *p*-*tert*-butylcalix[4]arene complex, with diethylether in the cavity; (**b**) Crystal structure of a triiridinum *p-tert-*butylcalix[5]arene complex with an encapsulated tetrafluoroborate anion inside [58]. Reprinted with permission from Staffilani, M.; Hancock, K.S.B.; Steed, J.W.; Holman, K.T.; Atwood, J.L.; Juneja, R.K.; Burkhalter, R.S. Anion binding within the cavity of π-metalated calixarenes *J. Am. Chem. Soc.* **1997**, *119*, 6324–6335. Copyright 1997 American Chemical Society.

Metal complexes are frequently bound to the outside of cavities, particularly with the electron-rich outside phenolic parts of calixarenes. For example, *p-tert*-butylcalix[4]arene coordinates rhodium outside, which allows to bind inside small neutral compounds such as diethylether or small anions such as tetrafluoroborate inside (Figure 14) [58].

Crystal structures of metal complexes with calix[4]arenes often show metal ions both in- and outside the cavity, e.g., with dinuclear Ti-IV and Ti-III complexes [59]. Calix[4]bisthiacrowns form with silver an endocyclic disilver complex and with copper exocyclic coordination polymers [60]. Stacking between the π-surfaces at the outside of 1,3-*bis*-pyridylmethylcalix[4]arene with different aryl compounds such as perfluoroarene or 1,4-dibromotetrafluorobenzene leads to infinite one-dimensional non-covalent ribbons [61].

**Figure 15:**
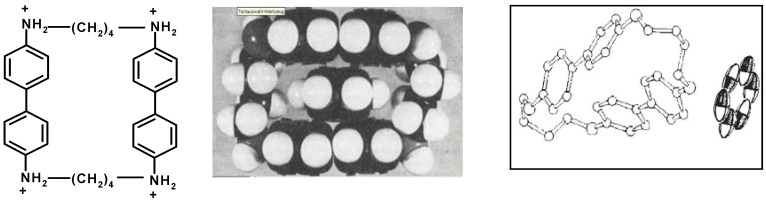
A benzidine-derived cyclophane and its complexation with benzene, expected from Corey–Pauling–Koltun (CPK)-model [62], and as seen in the crystal by X-ray [63]; in aqueous solution the benzene is inside [64]. Adapted from ref. [5] with permission from Wiley/VCH.

Larger cyclophanes of the type shown in Figure 15 are expected to bind phenyl derivatives in the cavity, as inferred early by Stetter *et al*. from the formation of a 1:1 crystalline complex with benzene, and from fitting to CPK models [62]. Later, however, X-ray analysis revealed that the Stetter crystal has the benzene located outside [63]. Many years later NMR-spectra showed that, in water, benzene in fact does bind within the cavity [64].

**Figure 16:**
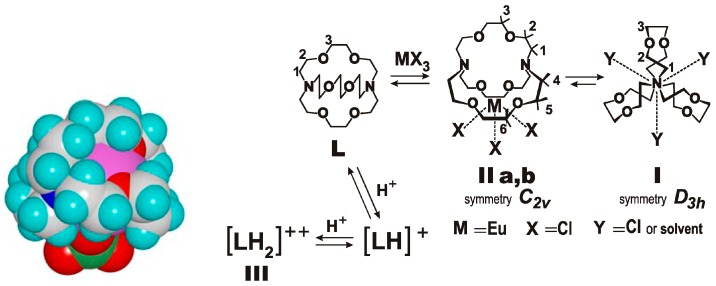
Complex of europium ion and a (222) cryptand, crystal structure with the metal ion inside [65] and structures with the metal in different locations, as observed in solution by NMR spectroscopy [66]. Partially reprinted from ref. [65] with permission of the Royal Society of Chemistry.

With a complex of europium ion and a (222) cryptand, one can observe the slow movement of the guest out of the cavity to the solution (Figure 16). If one dissolves the solid crystals, which from an earlier X-ray analysis is known to form as expected the inner sphere complex [65], in water (D_2_O) decomposition occurs into the free metal salt and the protonated ligand. Depending on the pH, two forms of metal complexes with different symmetry appear, as evident from the ^1^H-NMR spectra [66].

**Figure 17:**
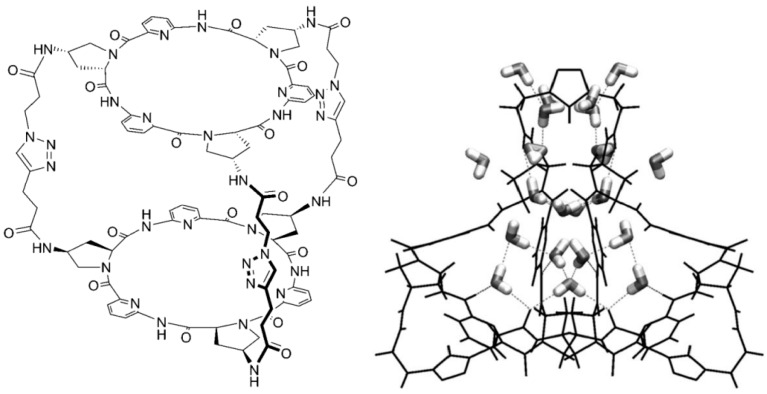
A triply linked bis-cyclopeptide complexing in aqueous solution with high efficiency sulfate ions inside the cavity; in the crystal (right side) only water, no sulfate, is found inside [67]. Adapted from ref. [67] with permission from Wiley/VCH.

The triply linked *bis*-cyclopeptide shown in Figure 17 exhibits remarkable differences between solution and solid state. In aqueous medium the host complexes a sulfate anion with lgK = 6, driven entirely by a gain in entropy. NMR data show that the sulfate is inside the cavity, forming hydrogen bonds to the amide NH groups at the inner surface of the host. In the crystal, however, one finds only water in the cavity, even though the crystals were grown in a solution containing sulfate [67].

Cyclodextrin complexes are prone to differ in the solid and solution state, since the hydrophobic effect as important driving force is missing in crystals, and the inside of cyclodextrins offers only C–H bonds for non-covalent interaction, in contrast to the outside and rim. Hydrophilic compounds are said to generally bind with cyclodextrins preferentially outside the cavity [68]; earlier publications suggested similar possibilities [69]. Open chain analogues of cyclodextrins often show even more efficient chromatographic enantiorecognition of e.g., drugs [70,71,72]. However there are until now not enough conclusive spectroscopic studies for related cyclodextrin complexes in the solid and solution state.

## 8. Cavity Filling Factors—Conflict with the Lock-and-Key Principle?

**Figure 18:**
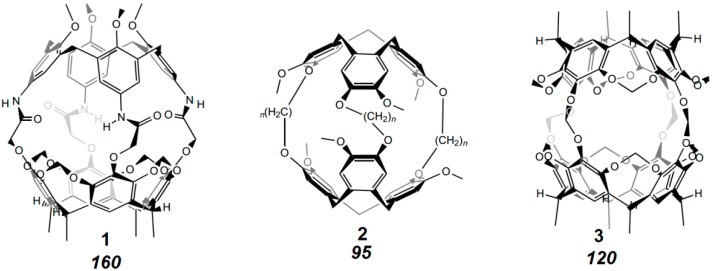
Calix[4]arene-carceplex **1**, cryptophane **2** (*n* = 2), and carceplex **3**, with volume of the internal cavity, in [Å^3^] [85].

Cyclophanes, cavitands and capsules have been shown to bind all kind of organic ligands, particularly those of an aromatic nature, in solution inside the cavity as long as the host leaves enough room for the guest molecule [73,74,75,76,77,78,79,80]. However, it has been noted early that there are deviations from the simple lock-and-key picture. Collet *et al.* found that water-soluble derivatives of cryptophanes, such as **2** in Figure 18, bind ammonium guest molecules in water not as expected as a function of the size match, but preferred a loose association with smaller ligands [81]. Similarly, fluorophores composed of smaller phenyl-parts and larger naphthyl-parts bind in water to cyclodextrins, not with the better fitting larger naphthyl part but with the seemingly too small phenyl entity [82].

Collet *et al.* showed already in 1993 [83,84] for cryptophanes such as **2** in Figure 18, that e.g., chloroform binds better than methane, although methane fits geometrically as well in the cavity; they calculated for CHCl_3_ in **2** an occupancy factor or packing coefficient (PC) of 0.886, corresponding to a very closely packed crystal; they also observed that the measured free enthalpy and entropy of complexation is comparable with the heat and entropy of crystallization of organic compounds. In contrast, for methane, PC amounts to only 0.348, which is consistent with later systematic evaluations by Rebek *et al.* [85] Analyses of many supramolecular complexes in solution, comprising in particular container- and capsule-type hosts have led Rebek *et al.* to the important generalization, that optimal binding occurs if 55% ± 9% of the space available in a cavity is occupied [86,87,88,89,90,91,92,93,94,95]. This is in the range of the packing density of organic liquids with a packing coefficient (PC) 0.51 to 0.63. Binding in hosts such as those in Figure 18 is indeed only observed if the PC is between 0.43 and 0.63. Larger packing coefficients of up to 0.70 can be reached if the complex is particularly stabilized by non-covalent interactions; in crystals and the interior of globular proteins the reported PC amounts to 0.66 to 0.77 [85].

That only a part of the available space is used for filling a cavity seems at first sight to be in conflict with the traditional lock-and-key principle. However, thermal motions, and the vibrational and translatory freedom of movement of host and guest require additional space. Moreover, a complete geometric match between host and guest molecules without any empty space between the complementary van der Waals surfaces can barely exist in the interaction between molecules of different shape and nature, characterized by corners and dimples. The exact calculation of the volume enclosed by the van der Waals surface is also therefore difficult, different methods can lead to variations of up to e.g., 15% [96]. Molecular dynamics (MD) simulation at 300 K predict e.g., that the volume in cavitands such as in Figure 18 vary over a range of 10% [85].

Polycyclic aromatic hydrocarbons (PAHs) with high binding affinities resulting from stacking and C–H···π interactions show larger deviation from Rebeks 55% filling factor [97]. Deviation from the optimal occupation rule was also observed e.g., with deep-cavity cavitand complexes in water [98]. A crystalline cryptophane complex with xenon exhibits an unusually large packing coefficient of 0.82 instead of 0.55 ± 0.09, with very short Xe···C contacts [99].

**Figure 19:**
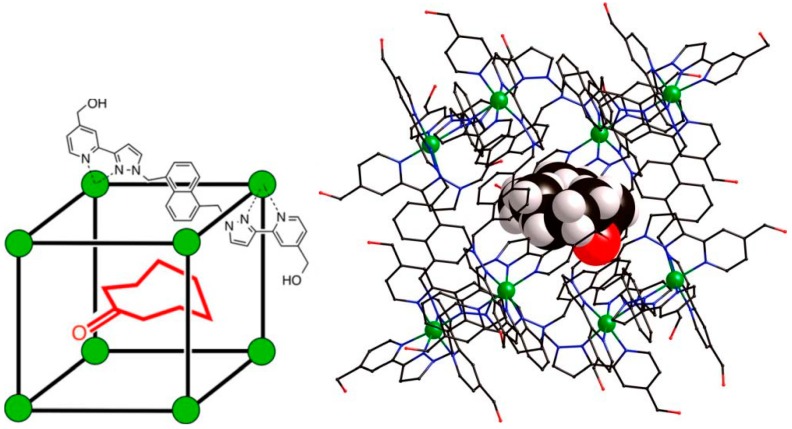
Host cage [Co_8_L_12_](BF_4_)_16_, complex with cycloundecanone, with a 55% occupancy of the cavity space, Co atoms in green [100]. With permission from Turega, S.; Cullen, W.; Whitehead, M.; Hunter, C.A.; Ward, M.D. Mapping the internal recognition surface of an octanuclear coordination cage using guest libraries *J. Am. Chem. Soc.* **2014**, *136*, 8475–8483. Copyright 2014 American Chemical Society.

Complexes of an octanuclear cubic coordination cage (Figure 19) in water with a series of aliphatic cyclic ketones show a linear relation between the guest’s surface and the binding ΔG as long as a 55% occupancy is reached [100]. Whether a crystal contains a guest molecule inside a host cavity can also depend on the preparation mode. With the complex shown in Figure 19 growing crystals from solvents containing excess guest afforded only the empty cage, whereas immersing preformed crystals of the cage in the neat guest cycloundecanone yielded the crystal with the entrapped guest [100].

## 9. Problems with Identification of Weak Interactions in Crystals

Crystallography has been the most important source for metrical details also of intermolecular bonds [101,102]. The availability of nearly half a million crystal structures in the Cambridge Structural Data Base (CSD) allows identification of the most significant non-covalent interactions also in supramolecular complexes with respect to their geometry [103]. The combination with computational approaches has led to often reliable generalizations also for weak interactions, although it has been stated that “experimentally found crystal structures of a given compound need not be those of minimal free energy” and that “the choice of relevant intermolecular bonds is sometimes arbitrary” [104]. This is different in solution or in the gas state: as long there is the commonly observed rapid exchange all occurring structures will reflect the dominating free energies.

That purely statistical evaluations with data bases such as the CSD can be misleading is obvious from the recent controversy about hydrogen bonds with organic fluorine as acceptor. Dunitz *et al.* found in 5947 crystal structures containing organic fluorine only 37%, or 0.6% with short CF···HX (X = O, N) distances, and therefore concluded in 2004 “Organic Fluorine Hardly Ever Makes Hydrogen Bonds” [105]. Other crystallographers did find evidence for hydrogen bonds with fluorine; e.g., Mehta and Sen [106] found with fluorinated polycyclitols H···F distances 2.55 Å and C–H···F angles around 150°; Desiraju *et al.* [107] found in layers of polyfluoro-substituted benzenes often 2.23–2.35 Å and C–H···F angles 150–175 Å; some researchers consider 2.41–2.78 Å H···F distances as still typical [108]. For other halogens (Cl, Br, I) crystal structures seemed to be in agreement with their possibility to act as hydrogen bond acceptor.

For solution and the gas state, all available evidence clearly speaks for fluorine as in fact a much better acceptor than other halogen derivatives [109], which in view of the electronegativity differences is of course expected in the framework of Pauling’s description of hydrogen bonds. In particular, measurements of equilibrium constants between compounds with a large range of donors and halogen acceptors in solvents such as CCl_4_ or CHCl_3_ furnished interaction free energies [109], systematically decreasing from e.g., 7.5 kJ/mol for fluoroalkanes RF to 4.7 kJ/mol for iodoalkanes RI (tested with 1-haloadamantanes with 4-fluorophenol in CCl_4_), with a systematical dependence on the substitution fragment for all halides [110]. For binding of fluoro derivatives to proteins, which is important in view of the 20% fluorine occurrence of all drugs, there is also clear indication of relatively strong hydrogen bonding with organic fluorine [111].

Obviously, the chances to find a significant number of hits in crystals of the thousands of fluorine containing compounds which have been prepared for all kind of reasons amounts to a lottery. The search for weak non-covalent forces in crystals is more promising if no other strong interactions are dominating the lattice: this is the case for example in pure hydrocarbons with e.g., one or more fluorine atoms, or if ones compares similar structures with many of the weak interactions one is looking for. Also, the search in protein databases is more promising, as generally protein complexes are more preselected—nobody will go to the expense of a solid state protein X-ray or NMR analysis if there is no prior evidence or at least hope that e.g., a fluorine generates a particular effect.

## 10. Conclusions

**Figure 20:**
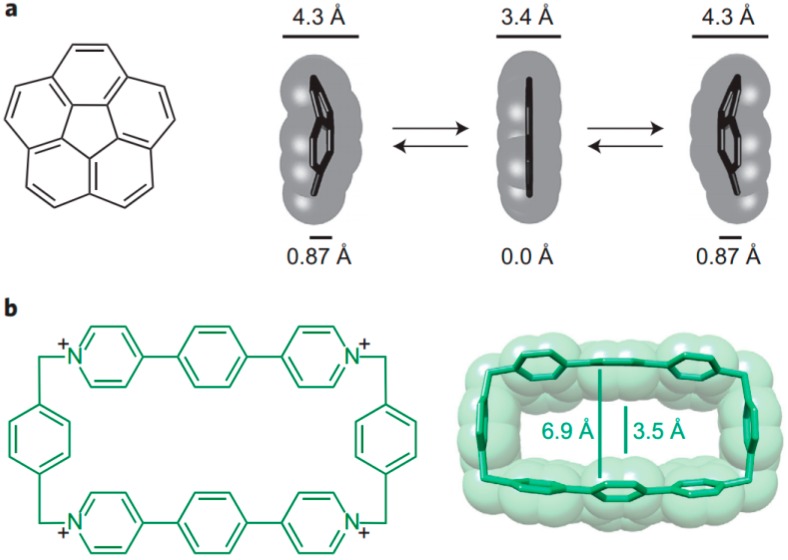
Corannulene (**a**) fits to a tetracationic cyclophane host (**b**) only in the flat transition state structure of the substrate, not its ground state, leading to faster inversion of the corannulene [114]. Reprinted from ref. [114] with permission from Nature Publishing Group.

The lock-and-key principle is still a valuable starting point for the understanding and the design of natural and synthetic supramolecular complexes. Recent examples show the importance of the lock-and-key principle and induced fit also for selectivity in enzymatic reactions [112,113]; how it can apply to the stabilization of transition states has been demonstrated with the bowl-to-bowl inversion of the non-planar corannulene by complexation with a tetracationic cyclophane, accompanied by an induced fit [114]. As illustrated in Figure 20 only the flat transition state structure of the substrate, not its ground state fits into the host cavity, which leads to a calculated rate increase of inversion by a factor of 10.

As demonstrated in this review the lock-and-key principle underlies important modifications. Optimal geometric fit may be a prerequisite, but high binding affinities depend often on a whole range of other factors, as discussed above. The possible self-inclusion of side groups is also a limitation of the simple lock-and-key concept, as are associations between several host molecules, in which one part of the host is inserted in the cavity of another one. Both interferences depend on the surrounding medium, and can in particular differ in the solid state. Typically, complexes in which the ligand occupies not the cavity of a host but are located outside are more often found in crystals than in solution. Statistical evidence from the analysis of not pre-selected crystal structure databases can be misleading with respect to the identification of very weak interactions. Structures of supramolecular complexes in solution can be evaluated by spectroscopic methods, preferably by NMR spectroscopy. The most often used Nuclear Overhauser Effect (NOE) provide intermolecular distances, but may reflect complexes which exhibit very short distances, and yet are less populated. In contrast to NOE data chemical shifts reflect usually the mixture of all conformers present in solution, according to their stability. Although the accuracy of structure elucidation based on chemical shifts cannot compete with crystallography they can be a useful and time-saving approach for the characterization of host–guest complexes. Both semiempirical and quantum-chemical calculations have been developed for this purpose [115,116,117,118], recently with emphasis on protein structures [119,120,121].
